# Integrating Proteome-Wide Association Studies and Single-Cell Transcriptomics Identifies GSTT2B as a Causal Mediator and Prioritizes COL4A1 in Diabetic Retinopathy

**DOI:** 10.3390/ijms27146178

**Published:** 2026-07-10

**Authors:** Lei Wen, Yuan Liu, Ka Zhang, Aiqin Mao, Li Geng, Fan Yu, Lei Feng, Hao Kan

**Affiliations:** 1 Wuxi School of Medicine, Jiangnan University, Wuxi 214126, China; 2 School of Food Science and Technology, Jiangnan University, Wuxi 214126, China

**Keywords:** diabetic retinopathy, PWAS, SMR, COL4A1, snRNA-seq

## Abstract

Diabetic retinopathy (DR) is a leading cause of vision loss, yet its systemic proteomic mediators remain largely elusive. This study aimed to identify causal plasma proteins, map their cell-type-specific localization in the retina, and experimentally validate their expression under disease-relevant stress. We conducted a proteome-wide association study (PWAS) integrating UK Biobank plasma pQTL data (N = 53,022) with DR GWAS summary statistics. Causal relationships were inferred utilizing summary-data-based Mendelian randomization (SMR) and Bayesian colocalization. Prioritized candidates were mapped to the Human and Mouse Retina Cell Atlases via single-nucleus RNA sequencing (snRNA-seq). Finally, to substantiate the computational findings, in vitro validation of *COL4A1* was performed in ARPE-19 cells cultured under hyperglycemic conditions utilizing quantitative real-time PCR (qPCR) and transcriptomic dataset re-analysis. The PWAS identified 26 proteins significantly associated with DR. Subsequent causal inference prioritized 12 high-confidence candidates, including GSTT2B, COL4A1, PAM, and GALNT3. Notably, GSTT2B emerged as a Tier-1 protective causal protein (Z = −3.609; P_SMR_ = 1.22 × 10^−4^). snRNA-seq mapping revealed that *GSTT2B* is robustly expressed in Müller glia and the retinal pigment epithelium (RPE), whereas *COL4A1* is prominently enriched in vascular compartments. These specific expression signatures exhibited partial conservation across species with notable cell-type specific variations. Crucially, in vitro validation confirmed that *COL4A1* mRNA expression is significantly upregulated under high-glucose stress. Furthermore, druggability analysis highlighted actionable targets, identifying GSTT2B as a highly probable causal mediator and COL4A1 as a prioritized candidate for structural intervention. This study provides robust genetic, single-cell, and experimental evidence implicating specific plasma proteins in DR pathogenesis. The identification of GSTT2B-mediated protective pathways and the hyperglycemia-induced upregulation of COL4A1 offer a high-resolution molecular atlas to guide drug repositioning and precision therapeutic strategies.

## 1. Introduction

Diabetic retinopathy (DR) is a leading cause of preventable blindness among the global working-age population. Driven by the worldwide diabetes epidemic, it represents a profound and growing public health challenge [1,2]. While traditionally defined by microvascular lesions—such as basement membrane thickening and blood–retinal barrier (BRB) breakdown—the pathophysiology of DR is now recognized as a complex interplay between microvascular dysfunction and retinal neurodegeneration [3,4]. Although anti-VEGF therapies have achieved clinical success, a substantial proportion of patients exhibit suboptimal responses. This highlights an urgent need to identify novel biomarkers and therapeutic targets capable of addressing the multifaceted nature of the disease [5,6].

The human plasma proteome is a critical reservoir of biological information. It reflects systemic physiological states and provides a high-resolution window into disease mechanisms [7,8]. In the context of DR, the early compromise of the blood–retinal barrier facilitates the bidirectional exchange of proteins between the systemic circulation and the retinal microenvironment, making plasma proteins highly relevant biological proxies for localized neurovascular pathology [9]. Circulating proteins act not only as diagnostic indicators but also as potential causal drivers or protectors during the progression of diabetic complications [10,11]. Recent advances in protein quantitative trait loci (pQTL) studies, notably the UK Biobank Pharma Proteomics Project (UKB-PPP) [12], have enabled large-scale investigations into the genetic determinants of the proteome. Integrating these proteomic insights with genome-wide association studies (GWAS) allows researchers to move beyond simple correlations. Utilizing methodologies like proteome-wide association studies (PWAS) and summary-data-based Mendelian randomization (SMR), we can now robustly infer causal relationships [13,14,15].

Nevertheless, significant gaps remain in our understanding of the proteomic architecture of DR. Previous studies have largely focused on single-omic layers or lacked cell-type-specific validation, leaving the cellular substrates of identified causal proteins elusive [16,17,18,19]. Although the gut-vascular axis and localized neurovascular unit (NVU) dysfunction have emerged as central hubs in DR, the specific proteins mediating these processes remain incompletely characterized, as does their conservation across species [20,21,22,23]. Furthermore, translating these genetic signals into actionable therapeutic strategies requires a systematic evaluation of their druggability within the specific context of retinal cellular architecture [24].

In the present study, we implemented a comprehensive, multi-stage framework to identify and prioritize causal plasma proteins for DR. Leveraging UKB-PPP proteomic data and DR GWAS summary statistics, we conducted a robust PWAS, complemented by SMR and Bayesian colocalization analysis. To bridge the gap between systemic protein levels and localized retinal pathology, we utilized the Human and Mouse Retina Cell Atlases (HRCA and MRCA) to map prioritized candidates to specific retinal cell types at single-cell resolution. Finally, we evaluated the translational potential of these findings through rigorous functional enrichment and druggability assessments. Our results prioritize GSTT2B and COL4A1 as key causal mediators, culminating in a high-resolution molecular atlas to guide the development of precision therapies for DR.

## 2. Results

**Figure 1:**
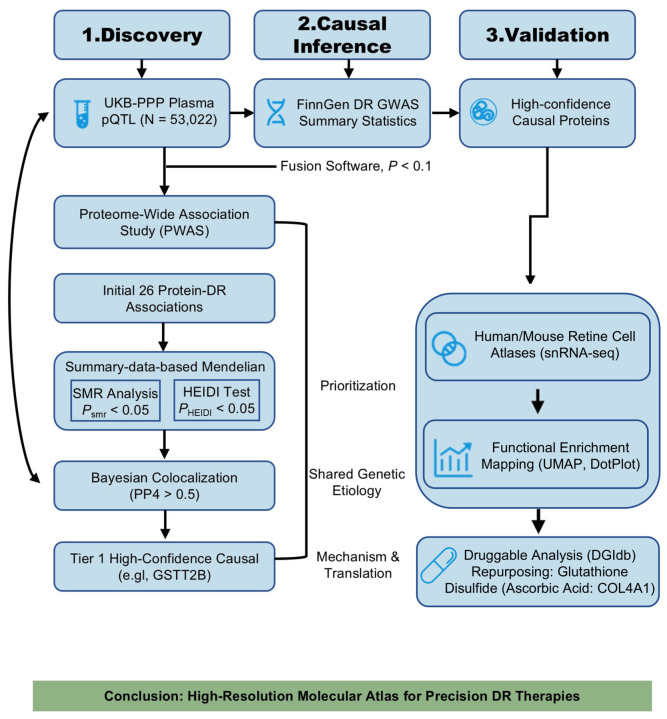
Multi-stage integrative framework for identifying and prioritization causal proteins for DR.

The overall study design is illustrated in Figure 1. We adopted a multi-stage integrative framework to identify putative causal proteins for DR. First, we performed a PWAS to detect statistically significant protein-trait associations. Subsequently, SMR and the HEIDI heterogeneity test were employed to infer causality and distinguish pleiotropy from linkage disequilibrium (LD). Finally, Bayesian colocalization analysis was conducted to validate the shared genetic architecture between plasma protein levels and DR risk. High-confidence causal proteins were subsequently prioritized for downstream functional annotation.

### 2.1. Identification of Plasma Proteomic Signatures for DR via PWAS

**Figure 2:**
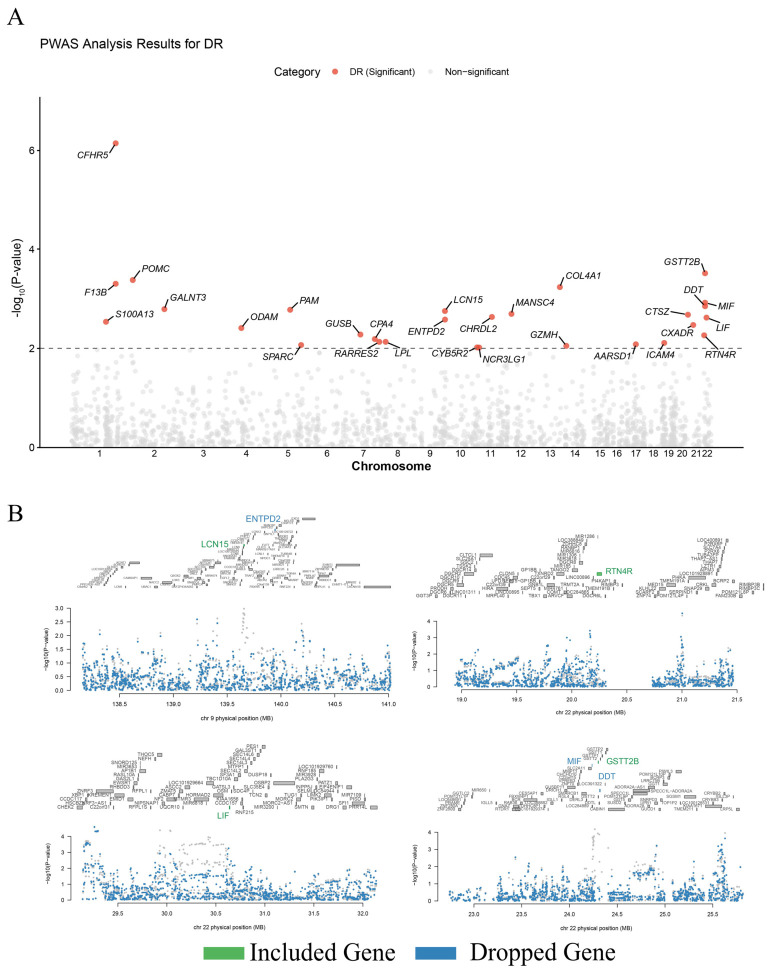
Results of the PWAS analyses. (**A**) Manhattan plot for the PWAS results for DR. The *x*-axis represents the chromosomal position, and the *y*-axis indicates the significance level (−log_10_*P* value). Significant genes passing the suggestive significance threshold (*p* < 0.01, dashed line) are highlighted in red and labeled with gene symbols. (**B**) Regional association plots for the identified risk loci following conditional analysis. The plots visualize the prioritization of causal genes within significant genomic regions. Genes highlighted in green (Included Gene) represent the prioritized candidates included in the credible set, while genes in blue (Dropped Gene) were filtered out due to high correlation (linkage disequilibrium) or lack of independent evidence.

We leveraged human plasma pQTL data from the UKB-PPP cohort (N = 53,022) to construct prediction models for 2923 proteins. Following rigorous quality control, 1715 proteins exhibiting significant SNP-based heritability (*p*  <  0.05, h^2^  >  0, Supplementary Table S1) were included in the PWAS. By integrating these proteomic data with DR GWAS summary statistics (Supplementary Table S2) via the FUSION pipeline, we initially identified 29 significant protein-DR associations (*p*  <  0.01; Figure 2A, Table 1, and Supplementary Table S3).

To resolve independent causal signals within these loci, we performed conditional analysis. This step filtered out three genes (*DDT*, *MIF*, and *ENTPD2*) due to LD contamination. Consequently, we obtained a final set of 26 unique genes significantly associated with DR (Figure 2B and Supplementary Table S4). Notably, CFHR5 demonstrated the most significant positive association (z = 4.957, *p* = 7.16 × 10^−7^), whereas GSTT2B exhibited a strong negative association (z = −3.609, *p* = 3.07 × 10^−4^).

### 2.2. Causal Prioritization via SMR and Bayesian Colocalization

**Figure 3:**
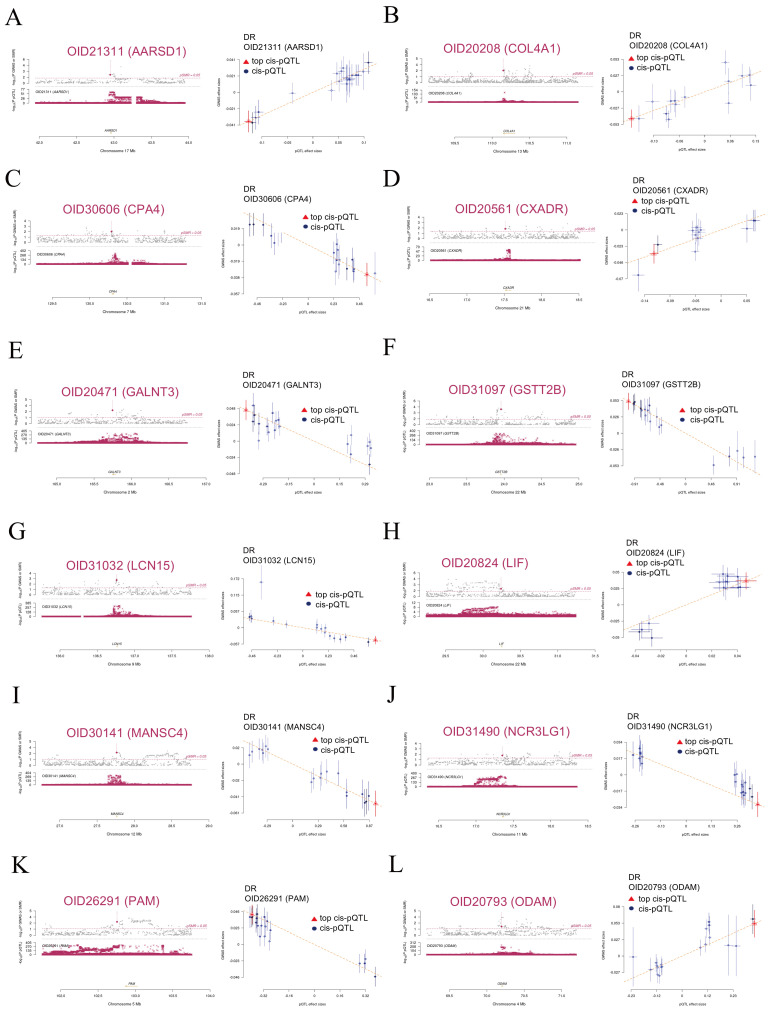
Results for the causal genes. SMR results for five putative causal proteins (**A**) AARSD1, (**B**) COL4A1, (**C**) CPA4, (**D**) CXADR, (**E**) GALNT3, (**F**) GSTT2B, (**G**) LCN15, (**H**) LIF, (**I**) MANSC4, (**J**) NCR3LG1, (**K**) PAM, and (**L**) ODAM. Left panels, regional association plots showing the colocalization of GWAS signals for DR (top plot, gray dots) and cis-pQTL signals for the respective plasma protein (bottom plot, maroon dots). The *x*-axis represents the genomic position, and the *y*-axis represents the −log_10_(*p*-value). The top SMR SNP is highlighted with a diamond. Right panels, effect size plots visualizing the relationship between the genetic effect on protein abundance (*x*-axis, cis-pQTL beta) and the genetic effect on DR risk (*y*-axis, GWAS beta). Each point represents a genetic instrument (SNP). The orange dashed line indicates the estimated causal effect (slope) from the SMR test. Error bars denote standard errors. Blue dots represent cis-pQTLs, and the red triangle highlights the top cis-pQTL instrument.

To robustly validate the causality of the 26 PWAS-identified candidates, we applied SMR analysis. While 17 proteins demonstrated significant associations in the SMR test (Supplementary Table S5), the heterogeneity in dependent instruments (HEIDI) test indicated that the signals for five of these targets were likely driven by LD rather than true causality (HEIDI *p* < 0.05). Consequently, 12 proteins (GSTT2B, GALNT3, PAM, LCN15, MANSC4, COL4A1, AARSD1, CPA4, LIF, CXADR, NCR3LG1, and ODAM) successfully passed both thresholds. This provides robust statistical evidence for their causal roles in DR (Figure 3A–L and Table 1).

**Figure 4:**
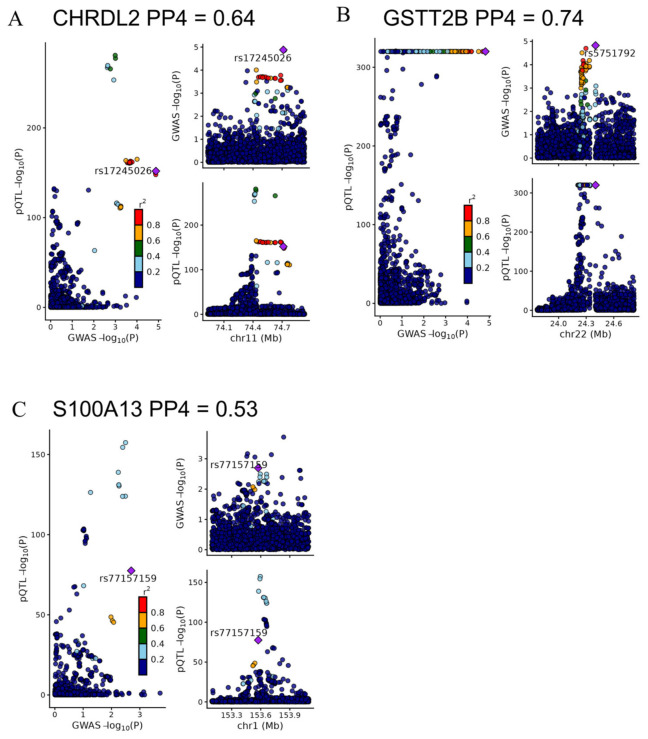
Bayesian colocalization analysis of DR GWAS signals and plasma pQTLs. LocusCompare plots visualizing the colocalization events for three significant genes (**A**) CHRDL2, (**B**) GSTT2B, and (**C**) S100A13. For each panel, the left plot shows the correlation between the significance of the GWAS association (*y*-axis, −log_10_*P*) and the pQTL association (*x*-axis, −log_10_*P*) for all variants in the region. The right plots display the regional Manhattan plots for GWAS (top) and pQTL (bottom) data. The purple diamond represents the lead SNP for each locus (labeled with rsID). Other SNPs are colored based on their linkage disequilibrium (r^2^) with the lead SNP. The posterior probability for a shared causal variant (PP4) is indicated above each panel, with a threshold of *PP4* > 0.5 considered suggestive evidence of colocalization.

To further rule out confounding by LD and confirm that the GWAS and pQTL signals share a single causal variant, we performed Bayesian colocalization analysis on the 26 candidates. Three proteins (GSTT2B, CHRDL2, and S100A13) exhibited suggestive evidence of colocalization, achieving a posterior probability of hypothesis 4 (*PP4*) > 0.5 (Figure 4A–C and Table 1).

Based on the cumulative evidence derived from the SMR, HEIDI, and colocalization analyses, we classified the candidate proteins into three tiers to systematically prioritize targets for functional follow-up. (1) Tier 1 (High confidence): One protein (GSTT2B) passed all three strict criteria (SMR significant, HEIDI non-significant, and Colocalization *PP4* > 0.5). (2) Tier 2 (Moderate confidence): Thirteen proteins (GALNT3, PAM, LCN15, MANSC4, CHRDL2, COL4A1, AARSD1, CPA4, LIF, CXADR, NCR3LG1, ODAM, and S100A13) demonstrated causal evidence in the SMR analysis but failed either the HEIDI test or the stringent colocalization threshold. (3) Tier 3 (Low confidence): Twelve proteins (CFHR5, F13B, CYB5R2, LPL, CTSZ, ICAM4, GUSB, GZMH, POMC, SPARC, RARRES2, and RTN4R) failed both the HEIDI test and colocalization analysis. This suggests that their associations are likely driven by complex LD structures at their respective genomic loci (Table 1).

### 2.3. Cell-Type-Specific Expression of DR-Associated Causal Genes in the Human and Mouse Retina

**Figure 5:**
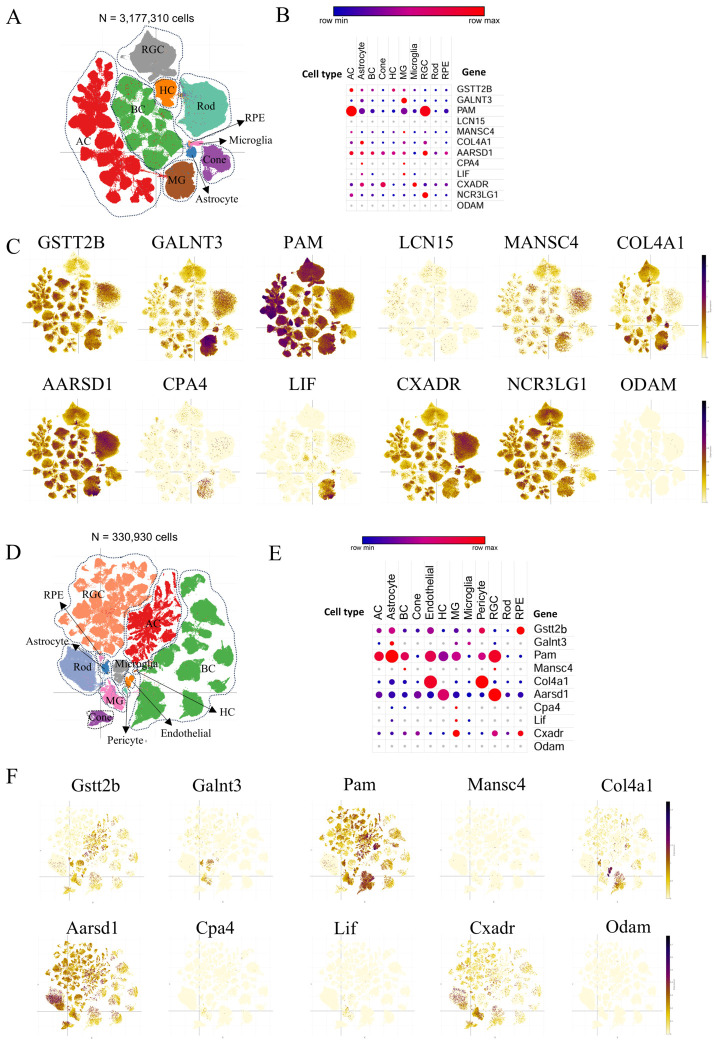
Single-cell transcriptomic profiling of putative DR causal genes. (**A**–**C**) Expression analysis in the human retina. (**A**) UMAP of 3,177,310 cells from the Human Retina Cell Atlas (HRCA), colored by major cell class. (**B**) Dot plot summarizing the expression of the twelve prioritized genes (*GSTT2B*, *GALNT3*, *PAM*, *LCN15*, *MANSC4*, *COL4A1*, *AARSD1*, *CPA4*, *LIF*, *CXADR*, *NCR3LG1*, *ODAM*) across human retinal cell types. The dot size represents the percentage of cells expressing the gene, and the color intensity (blue to red) indicates the average scaled expression level. (**C**) Feature plots visualizing the spatial expression patterns of the candidate genes on the human UMAP embedding. (**D**–**F**) Cross-species validation in the mouse retina. (**D**) UMAP visualization of 330,930 cells from the Mouse Retina Cell Atlas (MRCA). (**E**) Dot plot and (**F**) feature plots showing the expression of murine orthologs (*Gstt2b*, *Galnt3*, *Pam*, *Mansc4*, *Col4a1*, *Aarsd1*, *Cpa4*, *Lif*, *Cxadr*, *Odam*) in mouse retinal cell lineages. AC, Amacrine cells; BC, Bipolar cells; RGC, Retinal ganglion cells; HC, Horizontal cells; MG, Müller glia; RPE, Retinal pigment epithelium.

To determine the precise cellular localization of the 12 putative causal genes identified via PWAS and SMR (*GSTT2B*, *GALNT3*, *PAM*, *LCN15*, *MANSC4*, *COL4A1*, *AARSD1*, *CPA4*, *LIF*, *CXADR*, *NCR3LG1*, and *ODAM*), we evaluated their expression profiles using human retina single-nucleus RNA sequencing (snRNA-seq) data. Derived from the Human Retina Cell Atlas (HRCA, N = 3,177,310 cells; Figure 5A), the analysis clustered cells into 10 major types, including amacrine cells, astrocytes, bipolar cells, cone/rod photoreceptors, horizontal cells, Müller glia, microglia, retinal ganglion cells (RGCs), and retinal pigment epithelium (RPE).

Dot and feature plots revealed highly heterogeneous, cell-type-specific expression signatures for these 12 candidate genes (Figure 5B,C). Notably, *COL4A1* and *CXADR* exhibited prominent enrichment within the vascular and glial compartments, suggesting their critical roles in maintaining the BRB and overall structural integrity. *GSTT2B*, a top-tier candidate from our PWAS, was robustly expressed in Müller glia and RPE cells, pointing toward its potential involvement in metabolic support and antioxidant defense within the retinal microenvironment. Additionally, *PAM* and *GALNT3* demonstrated distinct expression patterns in neuronal lineages, particularly in amacrine and bipolar cells. This indicates that their contributions to DR pathogenesis may be mediated through NVU dysfunction.

To assess the evolutionary conservation of these localization patterns, we performed cross-species validation utilizing the Mouse Retina Cell Atlas (MRCA, N = 330,930 cells; Figure 5D). UMAPs and subsequent expression analyses of murine orthologs demonstrated partial concordance with the human dataset, alongside expected species-specific variations (Figure 5E,F). For instance, while *Col4a1* maintained highly consistent expression profiles within murine vascular cell populations, *Gstt2b* exhibited species-specific divergence, appearing highly expressed in the murine RPE. The identification of these conserved signatures across human and mouse retinas strengthens the functional relevance of these genes and strongly supports the utility of murine models for downstream mechanistic investigations. Collectively, these single-cell transcriptomic insights yield a high-resolution map of the cellular substrates through which these causal genes may modulate DR risk and progression.

### 2.4. Druggability Analysis of Prioritized Causal Proteins

To bridge the gap between genetic discovery and clinical application, we integrated our findings with drug-gene interaction databases to evaluate candidate protein druggability (Supplementary Table S6). This analysis identified several promising therapeutic avenues. GSTT2B, our core causal protein, is the pharmacological target of several approved therapeutic agents, including amitriptyline and glutathione disulfide. These compounds could potentially be repositioned for DR management leveraging their effects on metabolic and antioxidant pathways. Furthermore, COL4A1 and PAM interact with multiple approved compounds and nutraceuticals, such as ascorbic acid (vitamin C) and copper, both of which are critical for vascular basement membrane stability. Additionally, COL4A1 is targeted by proline and succinic acid, metabolites integral to tissue repair and collagen synthesis. These findings underscore the translational value of our genomic prioritization, highlighting both existing medications and nutritional interventions as viable strategies for precision therapy in DR.

### 2.5. Validation of COL4A1 Expression in a Hyperglycemic In Vitro Model

**Figure 6:**
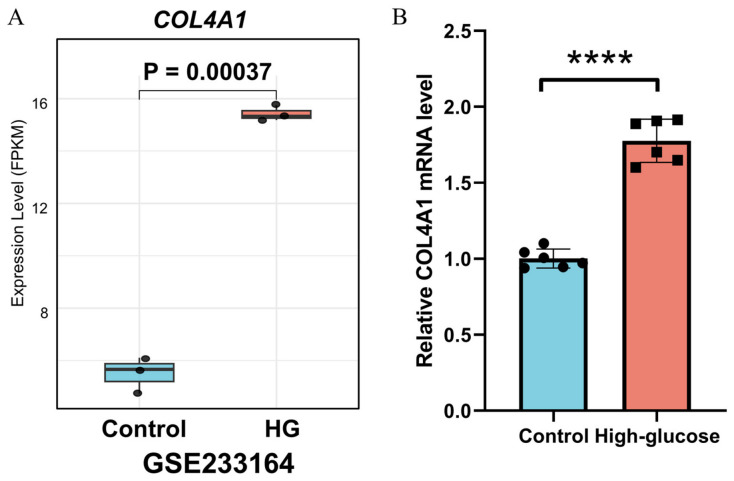
In vitro validation of *COL4A1* expression under hyperglycemic conditions. (**A**) Transcriptomic analysis of *COL4A1* expression in ARPE-19 cells based on the GSE233164 dataset. Cells were cultured in either normal glucose (Control) or high-glucose (HG) media for 72 h. (**B**) Quantitative real-time PCR (qPCR) validation of *COL4A1* mRNA expression in ARPE-19 cells under normal and high-glucose conditions. Data are presented as mean ± SD (n = 6). Statistical significance was determined by Student’s *t*-test. **** *p* < 0.0001 vs. Control group.

To substantiate the causal associations identified by our multi-omics pipeline, we evaluated the expression of *COL4A1* in an in vitro model of hyperglycemia, a primary metabolic driver of DR pathogenesis. First, we re-analyzed transcriptomic data from the GSE233164 dataset, which utilized ARPE-19 cells exposed to high-glucose media for 72 h. As illustrated in Figure 6A, *COL4A1* expression was significantly upregulated under high-glucose conditions compared to standard controls. Furthermore, to provide a comprehensive overview, the expression profiles of 10 prioritized causal genes under hyperglycemic stress in the GSE233164 dataset have been extracted and provided in Supplementary Table S7.

To independently corroborate these external findings, we performed qPCR analysis on ARPE-19 cells cultured under normal and high-glucose conditions. Consistent with the transcriptomic dataset, our experimental results demonstrated a significant increase in *COL4A1* mRNA levels in response to high glucose (Figure 6B). These in vitro findings align seamlessly with our computational predictions, experimentally confirming the dynamic upregulation of *COL4A1* under DR-relevant hyperglycemic stress.

## 3. Discussion

In this study, we employed an integrative multi-omics framework—combining PWAS, SMR, and single-cell transcriptomics—to systematically prioritize causal plasma proteins for DR. Our analysis identified 26 significant protein-DR associations, with GSTT2B emerging as a high-confidence (Tier 1) causal target. By mapping these associated genes to human and murine retinas at single-cell resolution, we localized their expression within the NVU, providing a molecular roadmap for potential therapeutic interventions.

A principal finding of our study is the robust negative association between GSTT2B levels and DR risk. GSTT2B (glutathione S-transferase theta-2B) is a key enzyme in the glutathione metabolism pathway, essential for neutralizing reactive oxygen species (ROS) [25,26]. Our single-cell analysis localized GSTT2B expression primarily to Müller glia and the RPE. In the context of DR, the retina undergoes chronic oxidative stress driven by hyperglycemia [27]. As Müller glia serve as the primary metabolic support cells of the retina, their robust expression of GSTT2B suggests a critical protective mechanism against glyoxidation-induced damage [28,29]. The negative Z-score observed in our PWAS indicates that genetically determined higher levels of GSTT2B are protective. This effect is potentially mediated by maintaining redox homeostasis within the retinal microenvironment and preventing the inflammatory cascades associated with glial activation.

Our pipeline also prioritized several targets involved in extracellular matrix (ECM) regulation and vascular integrity, most notably COL4A1 and CXADR. COL4A1 encodes the alpha-1 chain of type IV collagen, a major structural component of the vascular basement membrane. Furthermore, the identification of CXADR (coxsackievirus and adenovirus receptor) as a causal candidate is biologically noteworthy. Given that CXADR localizes to tight junctions [30,31]; its specific expression in retinal endothelial cells suggests that its dysregulation may directly contribute to the breakdown of the blood–retinal barrier (BRB), ultimately leading to clinical macular edema.

The robustness of our computational pipeline is further reinforced by in vitro validation in ARPE-19 cells. The observed upregulation of *COL4A1* under high-glucose stress aligns with the pathological ECM remodeling characteristic of early diabetic microvascular complications. Its hyperglycemia-induced overexpression typically contributes to basement membrane thickening, resulting in altered cellular interactions and vascular dysfunction in the retina. Together, these transcriptomic and experimental layers of evidence increase the biological plausibility of targeting the COL4A1 axis for DR intervention.

Traditionally viewed as a purely microvascular disease, DR is increasingly recognized as a complex neurovascular disorder [32,33]. Our findings support this paradigm, as causal targets such as PAM and GALNT3 exhibited distinct expression patterns within neuronal lineages, particularly amacrine and bipolar cells. PAM (peptidyl-alpha-hydroxyglycine alpha-amidating lyase) is essential for the activation of neuroendocrine peptides [34]. This finding underscores the necessity of developing therapeutic strategies that jointly target the vascular and neuronal compartments of the retina.

A key strength of the present study is the translational potential of our genetic findings. Our druggability analysis identified GSTT2B as a target for glutathione disulfide, reinforcing the clinical potential of antioxidant supplementation in DR management. Moreover, the established interaction between COL4A1 and ascorbic acid (vitamin C) provides a genetic rationale for nutritional interventions aimed at stabilizing the vascular basement membrane. Furthermore, the identification of approved drugs—such as amitriptyline—as potential GSTT2B modulators offers a compelling avenue for drug repositioning, which could significantly accelerate the development of novel DR therapies.

Despite the rigorous integrative approach employed, several limitations should be acknowledged. First, although we successfully validated the transcriptional upregulation of *COL4A1* using an external dataset and independent in vitro experiments, our wet-lab validation was performed in ARPE-19 epithelial cells. Because *COL4A1* is predominantly enriched in vascular endothelium, future studies should employ human retinal microvascular endothelial cells (HRMECs) to better recapitulate its cell-type-specific pathology. Second, due to reagent constraints, the exact functional response of the Tier-1 target, GSTT2B, requires dedicated experimental validation in future functional assays. Finally, the proteomic data utilized for the PWAS were derived from plasma. Although we leveraged retinal single-cell atlases to bridge the gap between systemic circulation and local tissues, future studies incorporating vitreous or retinal-specific proteomes will be necessary to achieve higher spatial resolution and tissue specificity.

## 4. Materials and Methods

### 4.1. Study Design and Integrative Framework

We implemented a multi-stage integrative genomic framework to systematically identify and prioritize causal plasma proteins for DR. The workflow proceeded through four synergistic phases. First, in the discovery phase, we conducted a PWAS to detect associations between genetically predicted protein abundance and DR risk. Second, for causal inference, we applied SMR and the HEIDI test to distinguish true pleiotropy from LD. Third, we performed Bayesian colocalization analysis to validate the shared genetic etiology between protein expression levels and DR risk. Finally, for functional characterization, we integrated snRNA-seq data from the Human and Mouse Retina Cell Atlases, subsequently performing pathway enrichment and druggability assessments to evaluate therapeutic potential.

### 4.2. Data Sources

#### 4.2.1. pQTL Data

Genetic instruments for plasma protein levels were obtained from the UK Biobank Pharma Proteomics Project (UKB-PPP) [12]. This cohort currently represents the largest proteomic dataset available, comprising 53,022 participants of European ancestry. Protein abundance was quantified using the Olink Explore 3072 platform, which is based on Proximity Extension Assay (PEA) technology and covers 2923 unique proteins. We utilized summary statistics for *cis*-pQTLs, defined as genetic variants located within ±500 kb of the transcription start site (TSS) of the corresponding protein-coding gene.

#### 4.2.2. GWAS for DR

Summary statistics for DR susceptibility were acquired from the FinnGen R12 release (https://www.finngen.fi/en, accessed on 10 June 2026) [35]. These data underwent standard quality control procedures. Specifically, variants with a minor allele frequency (MAF) < 0.01 or an imputation quality score < 0.3 were excluded from the downstream analysis.

### 4.3. Proteome-Wide Association Study (PWAS)

The PWAS was conducted utilizing the FUSION software pipeline (GitHub repository version, updated as of April 2026) [36]. First, we estimated the SNP-based heritability (h^2^) of each protein using the *cis*-pQTL data. Only proteins exhibiting significant heritability (*p* < 0.01) and a positive h^2^ were retained for predictive modeling. Genetic expression weights were subsequently computed using five distinct models (e.g., Elastic Net v1.5.2, LASSO v5, GBLUP v1.68, and BSLMM v1.0). For each protein, the model demonstrating the best cross-validation performance was selected. The resulting weights were integrated with the DR GWAS summary statistics to test the associations between genetically predicted protein levels and disease risk. Statistical significance was defined by a threshold of *p* < 0.01 [37]. To identify independent causal signals at genomic loci containing multiple significant proteins, we performed conditional and joint analyses using the GCTA-COJO tool (version v1.94.1) [38].

### 4.4. Causal Inference via SMR and HEIDI

To infer potential causal relationships, we applied the SMR method (version 1.32) [39,40]. This approach treats protein abundance as a mediator between genetic variants and DR, utilizing cis-pQTLs as instrumental variables. To distinguish true pleiotropy (where a single variant independently affects both protein levels and DR) from LD (where distinct, linked variants drive the observed associations), we performed the HEIDI test. A non-significant HEIDI result (*P*_HEIDI_ > 0.05) was required to support a robust causal model. For the prioritized PWAS candidates, we applied a significance threshold of *P*_SMR_ < 0.05.

### 4.5. Bayesian Colocalization Analysis

To provide stringent evidence of a shared causal variant, we performed Bayesian colocalization analysis utilizing the coloc R package (v4.6.3) [41]. For each genomic locus, we calculated the posterior probabilities (*PP*) across five mutually exclusive hypotheses. Our primary focus was PP4, which represents the probability that the pQTL and the DR GWAS signals share a single, identical causal variant. Given the distinct genetic architectures underlying plasma pQTLs and complex disease GWAS, a *PP4* > 0.5 was utilized as a threshold for suggestive colocalization evidence to prioritize candidates that had already cleared the SMR analysis.

### 4.6. Single-Cell Transcriptomic Analysis and Visualization

To map the cellular distribution of the prioritized causal genes, we accessed the Single Cell Portal hosted by the Broad Institute (https://singlecell.broadinstitute.org/single_cell, accessed on 10 June 2026.). We systematically queried two comprehensive reference atlases. First, we utilized the Human Retina Cell Atlas (HRCA; ‘HRCA: snRNA-seq of the human retina—all cells’ dataset). This dataset comprises over 3.1 million nuclei from 122 donors and was utilized to assess gene expression profiles within human retinal tissue. Second, to evaluate the evolutionary conservation of these expression patterns, we queried the Mouse Retina Cell Atlas (MRCA; ‘MRCA: scRNA-seq of the mouse retina—all cells’ dataset), which contains approximately 330,000 single cells from C57BL/6J mice. For both datasets, analyses were conducted utilizing the portal’s integrated interactive browser tools. Cell clusters were pre-annotated by the original authors based on established marker genes. We generated Uniform Manifold Approximation and Projection (UMAP) plots to visualize global cellular distributions. Additionally, dot plots were constructed to quantify both the expression intensity and the percentage of expressing cells for our candidate genes across the defined cell types. No secondary processing of raw data was performed; all visualizations accurately reflect the pre-processed and normalized data available on the portal.

### 4.7. Druggability Assessment

To evaluate the therapeutic potential of the identified causal proteins, we queried the Drug-Gene Interaction Database (DGIdb v5.0) [42] and the Open Targets Platform (https://platform.opentargets.org/, accessed on 10 June 2026) [43]. We specifically prioritized FDA-approved drugs and active investigational candidates. To ensure biological alignment, we cross-referenced each compound’s mechanism of action (e.g., inhibitor versus activator) with the direction of effect (β coefficient) derived from our PWAS and SMR analyses.

### 4.8. Re-Analysis of GEO Dataset

The dataset GSE233164 was acquired from the Gene Expression Omnibus (GEO) database. Normalized gene expression data were extracted for ARPE-19 cells cultured in either standard or high-glucose media for 72 h. The expression levels of COL4A1 were then compared between these experimental groups using Student’s *t*-tests to statistically evaluate its transcriptional response to hyperglycemic stress.

### 4.9. Cell Culture and Quantitative Real-Time PCR (qPCR)

Human retinal pigment epithelial cells (ARPE-19 [ATCC, Manassas, VA, USA]) were cultured in Dulbecco’s Modified Eagle Medium (DMEM) supplemented with 10% fetal bovine serum and 1% penicillin-streptomycin. To establish the in vitro hyperglycemia model, cells were incubated in media containing either standard glucose (5.5 mM) or high glucose (30 mM D-glucose) concentrations for 72 h. Total RNA was extracted using TRIzol reagent (CWBIO, Taizhou, China) according to the manufacturer’s protocol. This was followed by reverse transcription utilizing a commercial cDNA synthesis kit (Vazyme, Nanjing, China). Quantitative real-time PCR (qPCR) was performed using SYBR Green master mix (Vazyme, Nanjing, China) on an ABI 7500 Real-Time PCR System. Relative mRNA expression levels were calculated using the 2^−ΔΔct^ method. GAPDH served as the specific internal reference control for normalization. The precise primer sequences utilized in this study are detailed in Supplementary Table S8.

## 5. Conclusions

In summary, our study integrates genetic, proteomic, and single-cell transcriptomic data to identify a suite of causal proteins for DR. The discovery of the GSTT2B-mediated antioxidant pathway and the structural role of COL4A1 provides new insights into DR pathogenesis and highlights several druggable targets for future clinical trials.

## Figures and Tables

**Table 1:** Summary Results From PWAS, Colocalization, and SMR for 26 PWAS-Identified Proteins.

Protein	Protein Full Name	PWAS		Conditional		Colocalization	SMR			Category
		PWAS.Z	PWAS.P	JOINT.Z	JOINT.P	PP4 > 0.5	Beta_SMR	P_SMR	P_HEIDI_SMR	
CFHR5	Complement factor H-related protein 5	4.957	7.16 × 10^−7^	4.3	0.00002	NO	0.180511	8.72 × 10^−7^	2.75 × 10^−6^	tier3
GSTT2B	Glutathione S-transferase theta-2B	−3.609	0.000307	−3.6	0.00031	YES	−0.0506635	1.22 × 10^−4^	7.31 × 10^−1^	tier1
F13B	Coagulation factor XIII B chain	3.481	0.0005	−2.4	0.01652	NO	0.0952091	4.89 × 10^−4^	2.00 × 10^−6^	tier3
GALNT3	Polypeptide N-acetylgalactosaminyltransferase 3	−3.152	0.001622	−3.2	0.00162	NO	−0.116647	1.66 × 10^−3^	6.05 × 10^−2^	tier2
PAM	Peptidyl-alpha-hydroxyglycine alpha-amidating lyase	−3.144	0.00167	−3.1	0.0017	NO	−0.106338	1.80 × 10^−3^	3.35 × 10^−1^	tier2
LCN15	Lipocalin-15	−3.126	0.00177	−3.1	0.0018	NO	−0.0665664	1.82 × 10^−3^	3.74 × 10^−1^	tier2
MANSC4	MANSC domain containing 4	−3.087	0.00202	−3.1	0.002	NO	−0.0524078	1.98 × 10^−3^	9.54 × 10^−1^	tier2
CHRDL2	Chordin-like protein 2	−3.044	0.00233	−3	0.0023	YES	−0.151132	2.40 × 10^−3^	7.23 × 10^−3^	tier2
COL4A1	Collagen alpha-1(IV) chain	3.4388	0.000584	3.4	0.00058	NO	0.238567	2.79 × 10^−3^	5.03 × 10^−1^	tier2
CYB5R2	NADH-cytochrome b5 reductase 2	2.59105	0.00957	2.6	0.0096	NO	0.173264	6.35 × 10^−3^	5.38 × 10^−3^	tier3
AARSD1	Alanyl-tRNA editing protein Aarsd1	2.6404	0.00828	2.6	0.0083	NO	0.279238	7.31 × 10^−3^	9.78 × 10^−1^	tier2
LPL	Lipoprotein lipase	−2.679	0.00738	−2.7	0.0074	NO	−0.23187	7.93 × 10^−3^	1.73 × 10^−2^	tier3
CPA4	Carboxypeptidase A4	−2.722	0.00649	−2.7	0.0065	NO	−0.0651783	9.54 × 10−3	5.92 × 10−1	tier2
LIF	Leukemia inhibitory factor	3.035	0.002402	3	0.00241	NO	0.82493	1.02 × 10−2	3.27 × 10^−1^	tier2
CXADR	Coxsackievirus and adenovirus receptor	2.93132	0.00338	2.9	0.0034	NO	0.276286	1.45 × 10^−2^	5.32 × 10^−1^	tier2
NCR3LG1	Natural cytotoxicity triggering receptor 3 ligand 1	−2.589	0.00963	−2.6	0.0096	NO	−0.0856406	1.67 × 10^−2^	4.00 × 10^−1^	tier2
ODAM	Odontogenic ameloblast-associated protein	2.8872	0.00389	2.9	0.0039	NO	0.160525	1.69 × 10^−2^	2.45 × 10^−1^	tier2
CTSZ	Cathepsin Z	3.0764	0.0021	3.1	0.0021	NO	0.231579	7.21 × 10^−2^	7.22 × 10^−1^	tier3
S100A13	Protein S100-A13	−2.9772	0.00291	−3	0.00291	YES	−0.144997	9.56 × 10^−2^	6.21 × 10^−1^	tier2
ICAM4	Intercellular adhesion molecule 4	2.6618	0.00777	2.7	0.0078	NO	0.223454	1.07 × 10^−1^	8.11 × 10^−1^	tier3
GUSB	Beta-glucuronidase	−2.79044	0.00526	−2.8	0.0053	NO	−0.135204	2.06 × 10^−1^	4.66 × 10^−1^	tier3
GZMH	Granzyme H	−2.616191	0.00889	−2.6	0.0089	NO	−0.0720502	4.34 × 10^−1^	6.07 × 10^−1^	tier3
POMC	Corticotropin-like intermediary peptide	−3.5269	0.000421	−3.5	0.00042	NO	−0.174224	4.79 × 10^−1^	4.75 × 10^−1^	tier3
SPARC	SPARC	−2.629274	0.00856	−2.6	0.0086	NO	−0.0787028	7.13 × 10^−1^	3.70 × 10^−1^	tier3
RARRES2	Retinoic acid receptor responder protein 2	−2.67893	0.00739	−2.7	0.0074	NO	-	-	-	tier3
RTN4R	Reticulon-4 receptor	2.779	0.005453	2.8	0.00545	NO	-	-	-	tier3

PP4 > 0.5 means 2 signals were considered to have a strong support of colocalization. PWAS.Z: Z-score from discovery PWAS analysis; PWAS.P: *p*-value from discovery PWAS analysis; JOINT.Z: Z-score from conditional analysis; JOINT.P: *p*-value from conditional analysis; Beta_SMR: Effect estimate from the top snp SMR analysis; P_SMR: *p*-value from the top snp SMR analysis; P_HEIDI_SMR: *p*-value from HEIDI test.

## Data Availability

Publicly available datasets were analyzed in this study. This data can be found here: The GWAS summary statistics for DR based on FinnGen Consortium can be accessed at https://www.finngen.fi/en (accessed on 10 June 2026). The pQTL data can be accessed at https://registry.opendata.aws/ukbppp/ (accessed on 10 June 2026). The single-cell RNA sequencing data of HARC (project number: SCP2805) or MARC (project number: SCP2560) can be accessed at https://singlecell.broadinstitute.org/single_cell (accessed on 10 June 2026).

## Supplementary Materials

The following supporting information can be downloaded at: https://www.mdpi.com/article/10.3390/ijms27146178/s1.

## Abbreviations

The following abbreviations are used in this manuscript:

DR	Diabetic retinopathy
GWAS	Genome-Wide Association Studies
HEIDI	Heterogeneity in dependent instruments
HG	High glucose
LD	Linkage disequilibrium
SMR	Summary-data-based Mendelian randomization
PPI	Protein–protein interaction
pQTL	Proteomic quantitative trait loci
PWAS	Proteome-Wide Association Study
RPE	Retinal pigment epithelium
SNP	Single-nucleotide polymorphism
UKB-PPP	UK Biobank Pharma Proteomics Project
VEGF	Vascular endothelial growth factor

## References

[1] Wong T.Y., Cheung C.M., Larsen M., Sharma S., Simo R. (2016). Diabetic retinopathy. Nat. Rev. Dis. Primers.

[2] Sivaprasad S., Wong T.Y., Gardner T.W., Sun J.K., Bressler N.M. (2025). Diabetic retinal disease. Nat. Rev. Dis. Primers.

[3] Simó R., Hernández C., Frontoni S., Sbraccia P., Schlingemann R., Valldeperas X., Vujosevic S., Marques I., Cunha-Vaz J., Grauslund J. (2026). Relationship between retinal neurodysfunction and cognitive impairment in type 2 diabetes: Results of the RECOGNISED cross-sectional study. Diabetologia.

[4] Chen Y., Wang R., Zhang N., Xu L. (2025). Ferroptosis-Mediated Cell-Specific Damage: Molecular Cascades and Therapeutic Breakthroughs in Diabetic Retinopathy. Antioxidants.

[5] Ng D.S.C., Ruamviboonsuk P., Apte R.S., Bajimaya S., Chan C.K.M., Chang A., Cheung C.Y., Chen S.J., Chaudhary V., Chaikitmongkol V. (2025). International consensuses and controversies on causes, diagnosis and management of diabetic macular edema (DME). Prog. Retin. Eye Res..

[6] Levine S.R., Sapieha P., Dutta S., Sun J.K., Gardner T.W. (2022). It is time for a moonshot to find “Cures” for diabetic retinal disease. Prog. Retin. Eye Res..

[7] Zhang K., Wang P., Huang W., Tang S.H., Xue H., Wu H., Zhang Y., Rong Y., Dong S.S., Chen J.B. (2024). Integrated landscape of plasma metabolism and proteome of patients with post-traumatic deep vein thrombosis. Nat. Commun..

[8] Si S., Liu H., Xu L., Zhan S. (2024). Identification of novel therapeutic targets for chronic kidney disease and kidney function by integrating multi-omics proteome with transcriptome. Genome Med..

[9] Li H., Zhu Z., Yang S., Cheng W., Tan S., Xin Z., Zhang L., Zhu Z., Chen S., Huang W. (2026). Plasma proteomic signatures of early retinal neurodegeneration in diabetes: A multi-cohort study. PLoS Med..

[10] Li R., Tian S., Liu J., Li R., Zhu K., Lu Q., Qiu Z., Yu H., Li L., Franco O.H. (2025). Modifiable risk factors and plasma proteomics in relation to complications of type 2 diabetes. Nat. Commun..

[11] Yuan S., Xu F., Li X., Chen J., Zheng J., Mantzoros C.S., Larsson S.C. (2023). Plasma proteins and onset of type 2 diabetes and diabetic complications: Proteome-wide Mendelian randomization and colocalization analyses. Cell Rep. Med..

[12] Sun B.B., Chiou J., Traylor M., Benner C., Hsu Y.H., Richardson T.G., Surendran P., Mahajan A., Robins C., Vasquez-Grinnell S.G. (2023). Plasma proteomic associations with genetics and health in the UK Biobank. Nature.

[13] Friligkou E., Løkhammer S., Cabrera-Mendoza B., Shen J., He J., Deiana G., Zanoaga M.D., Asgel Z., Pilcher A., Di Lascio L. (2024). Gene discovery and biological insights into anxiety disorders from a large-scale multi-ancestry genome-wide association study. Nat. Genet..

[14] Xu F., Yu E.Y., Cai X., Yue L., Jing L.P., Liang X., Fu Y., Miao Z., Yang M., Shuai M. (2023). Genome-wide genotype-serum proteome mapping provides insights into the cross-ancestry differences in cardiometabolic disease susceptibility. Nat. Commun..

[15] Wingo A.P., Liu Y., Gerasimov E.S., Gockley J., Logsdon B.A., Duong D.M., Dammer E.B., Robins C., Beach T.G., Reiman E.M. (2021). Integrating human brain proteomes with genome-wide association data implicates new proteins in Alzheimer’s disease pathogenesis. Nat. Genet..

[16] Chen C., Zhang H., Lan Y., Yan W., Liu S., Chen Y., Xie T., Ning J., Yan X., Shang L. (2024). Statins as a risk factor for diabetic retinopathy: A Mendelian randomization and cross-sectional observational study. J. Transl. Med..

[17] Zheng D., Li N., Hou R., Zhang X., Wu L., Sundquist J., Sundquist K., Ji J. (2023). Glucagon-like peptide-1 receptor agonists and diabetic retinopathy: Nationwide cohort and Mendelian randomization studies. BMC Med..

[18] Zhang Z., Xie Y., Bu Z., Xiang Y., Sheng W., Cao Y., Lian L., Zhang L., Qian W., Ji G. (2025). Genetically proxied glucokinase activation and risk of diabetic complications: Insights from phenome-wide and multi-omics mendelian randomization. Diabetes Res. Clin. Pract..

[19] Stanhope S.C., Brandwine-Shemmer T., Blum H.R., Doud E.H., Jannasch A., Mosley A.L., Minke B., Weake V.M. (2023). Proteome-wide quantitative analysis of redox cysteine availability in the Drosophila melanogaster eye reveals oxidation of phototransduction machinery during blue light exposure and age. Redox Biol..

[20] Wang R., Wang Q.Y., Bai Y., Bi Y.G., Cai S.J. (2023). Research progress of diabetic retinopathy and gut microecology. Front. Microbiol..

[21] Alarcón Yempén R.E., Venzel R., Paulino Campos M.C., de Oliveira L.P., Lins R.V.D., Pessoni A.M., Fanaro G.B., de Oliveira Souza A., Calaza K.D.C., de Brito Alves J.L. (2021). Gut microbiota: A potential therapeutic target for management of diabetic retinopathy?. Life Sci..

[22] Sinclair S.H., Schwartz S. (2024). Diabetic retinopathy: New concepts of screening, monitoring, and interventions. Surv. Ophthalmol..

[23] Nian S., Lo A.C.Y., Mi Y., Ren K., Yang D. (2021). Neurovascular unit in diabetic retinopathy: Pathophysiological roles and potential therapeutical targets. Eye Vis..

[24] Xue Z., Yuan J., Chen F., Yao Y., Xing S., Yu X., Li K., Wang C., Bao J., Qu J. (2022). Genome-wide association meta-analysis of 88,250 individuals highlights pleiotropic mechanisms of five ocular diseases in UK Biobank. eBioMedicine.

[25] Butler M.W., Hackett N.R., Salit J., Strulovici-Barel Y., Omberg L., Mezey J., Crystal R.G. (2011). Glutathione S-transferase copy number variation alters lung gene expression. Eur. Respir. J..

[26] Wang T., Weng M., Li K., Li G., Hu S., Hu Z., Li Y., Li M., Wu D., Liang Z. (2025). LIN28B enhances the chemosensitivity of colon cancer cells via inducing genomic instability by upsetting the balance between the production and removal of reactive oxygen species. Cancer Lett..

[27] Yang J., Liu Z. (2022). Mechanistic Pathogenesis of Endothelial Dysfunction in Diabetic Nephropathy and Retinopathy. Front. Endocrinol..

[28] Hoang T., Wang J., Boyd P., Wang F., Santiago C., Jiang L., Yoo S., Lahne M., Todd L.J., Jia M. (2020). Gene regulatory networks controlling vertebrate retinal regeneration. Science.

[29] Trevino A.E., Müller F., Andersen J., Sundaram L., Kathiria A., Shcherbina A., Farh K., Chang H.Y., Pașca A.M., Kundaje A. (2021). Chromatin and gene-regulatory dynamics of the developing human cerebral cortex at single-cell resolution. Cell.

[30] Kwon J.W., Kim N.H., Choi I. (2016). CXADR is required for AJ and TJ assembly during porcine blastocyst formation. Reproduction.

[31] Nilchian A., Johansson J., Ghalali A., Asanin S.T., Santiago A., Rosencrantz O., Sollerbrant K., Vincent C.T., Sund M., Stenius U. (2019). CXADR-Mediated Formation of an AKT Inhibitory Signalosome at Tight Junctions Controls Epithelial-Mesenchymal Plasticity in Breast Cancer. Cancer Res..

[32] Antonetti D.A., Silva P.S., Stitt A.W. (2021). Current understanding of the molecular and cellular pathology of diabetic retinopathy. Nat. Rev. Endocrinol..

[33] Tang L., Xu G.T., Zhang J.F. (2023). Inflammation in diabetic retinopathy: Possible roles in pathogenesis and potential implications for therapy. Neural Regen. Res..

[34] Chen Y.C., Taylor A.J., Verchere C.B. (2018). Islet prohormone processing in health and disease. Diabetes Obes. Metab..

[35] Kurki M.I., Karjalainen J., Palta P., Sipilä T.P., Kristiansson K., Donner K.M., Reeve M.P., Laivuori H., Aavikko M., Kaunisto M.A. (2023). FinnGen provides genetic insights from a well-phenotyped isolated population. Nature.

[36] Wang J.H., Dong S.S., Huang W., Wang H.A., Liu S.S., Ma X., Zhu R.J., Shi W., Wu H., Yu K. (2025). Blood plasma proteome-wide association study implicates novel proteins in the pathogenesis of multiple cardiovascular diseases. Cardiovasc. Diabetol..

[37] Brandes N., Linial N., Linial M. (2020). PWAS: Proteome-wide association study-linking genes and phenotypes by functional variation in proteins. Genome Biol..

[38] Restuadi R., Steyn F.J., Kabashi E., Ngo S.T., Cheng F.F., Nabais M.F., Thompson M.J., Qi T., Wu Y., Henders A.K. (2022). Functional characterisation of the amyotrophic lateral sclerosis risk locus GPX3/TNIP1. Genome Med..

[39] Lin P.W., Lin Z.R., Wang W.W., Guo A.S., Chen Y.X. (2025). Identification of immune-inflammation targets for intracranial aneurysms: A multiomics and epigenome-wide study integrating summary-data-based Mendelian randomization, single-cell-type expression analysis, and DNA methylation regulation. Int. J. Surg..

[40] Zhang K., Liu Y., Mao A., Li C., Geng L., Kan H. (2025). Proteome-Wide Mendelian Randomization Identifies Therapeutic Targets for Abdominal Aortic Aneurysm. J. Am. Heart Assoc..

[41] Lin J., Zhou J., Xu Y. (2023). Potential drug targets for multiple sclerosis identified through Mendelian randomization analysis. Brain.

[42] Cannon M., Stevenson J., Stahl K., Basu R., Coffman A., Kiwala S., McMichael J.F., Kuzma K., Morrissey D., Cotto K. (2024). DGIdb 5.0: Rebuilding the drug-gene interaction database for precision medicine and drug discovery platforms. Nucleic Acids Res..

[43] Ochoa D., Hercules A., Carmona M., Suveges D., Baker J., Malangone C., Lopez I., Miranda A., Cruz-Castillo C., Fumis L. (2023). The next-generation Open Targets Platform: Reimagined, redesigned, rebuilt. Nucleic Acids Res..

